# Childhood Trauma and Non-suicidal Self-Injury Among Chinese Adolescents: The Mediating Role of Psychological Sub-health

**DOI:** 10.3389/fpsyt.2022.798369

**Published:** 2022-02-10

**Authors:** Cui Huang, Qiuyu Yuan, Menglin Ge, Xuanlian Sheng, Meng Yang, Shengya Shi, Panpan Cao, Mengting Ye, Ran Peng, Ruochen Zhou, Kai Zhang, Xiaoqin Zhou

**Affiliations:** ^1^Chaohu Hospital, Anhui Medical University, Hefei, China; ^2^School of Mental Health and Psychological Sciences, Anhui Medical University, Hefei, China; ^3^Anhui Psychiatric Center, Anhui Medical University, Hefei, China

**Keywords:** non-suicidal self-injury, adolescent, psychological sub-health, childhood trauma, left-behind children

## Abstract

The factors associated with non-suicidal self-injury (NSSI) of adolescents have been widely researched. However, the underlying mechanism of the relationship between childhood trauma and NSSI is limited. This study aimed to explore the risk factors for NSSI among Chinese adolescents. Our hypothesis was that psychological sub-health (PSH) played a mediating role between childhood trauma and NSSI. The Childhood Trauma Questionnaire, the Multidimensional Sub-health Questionnaire of Adolescent, and the self-report NSSI were used to measure childhood trauma, PSH, and NSSI. Structural equation model (SEM) was performed to verify our hypothesis. The results showed that 33.9% of the participants in our survey had engaged in NSSI in the past year. Adolescents who were left-behind children or in primary schools were more likely to engage in NSSI. Additionally, 56.2% of the participants had moderate to severe childhood trauma, and 26.1% of the participants had PSH. Furthermore, childhood trauma and PSH would increase the risk of NSSI by 2 times (B = 0.79, *p* < 0.01) and 5 times (B = 1.64, *p* < 0.01), respectively. SEM was established (*p* = 0.512) and the goodness-of-fit indices were examined (CMIN/DF = 0.892; GFI = 0.997; AGFI = 0.992; NFI = 0.991; RFI = 0.980; IFI = 1.00; TLI = 1.00; CFI = 1.00; RMSEA < 0.001). The SEM indicated that childhood trauma positively predicted NSSI both directly and indirectly through PSH. PSH has been confirmed to have partial mediating effects between childhood trauma and NSSI. The assessment of PSH may be an operable and effective method to screen and predict NSSI. Meanwhile, the intervention of childhood trauma and PSH may effectively prevent and reduce the occurrence of NSSI among adolescents.

## Introduction

Non-suicidal self-injury (NSSI) is usually defined as deliberate and self-inflicted damage to the body without suicidal intent, which excludes socially accepted behaviors (e.g., piercing, tattooing, or religious rituals) (1). The essential difference between NSSI and suicide, suicidal ideation or suicide attempts is that NSSI is defined as occurring without suicidal intent. Yet another widely used term for nonfatal self-mutilation is self-harm (regardless of suicidal intent), and the wish to die may be one of the motives or reasons for self-harm. NSSI is an urgent public health problem. The global prevalence of NSSI among adolescents is estimated to be 17.2% (2), while that in China is 22.37% (3). Self-injurious thoughts and behaviors are risk factors for further suicidal ideation, attempts, and death (4–6). More than 800 000 people die by suicide every year (7), while each suicide in a population is accompanied by more than 20 suicide attempts (8). In addition, NSSI was significantly and prospectively associated with increased levels of suicide ideation and suicide attempts and was identified as an important factor to assess the risk for later suicidality (9). Analysis of global patterns of mortality in young people indicated that NSSI has been verified as the strongest predictor of future suicide (10).

The COVID-19 pandemic poses greater challenges to mental health, however, research on NSSI among adolescents was limited (11). An emergency department in Ireland observed a reduction (35%) initially and followed a sharp increase (104%) in patients with self-harm from March to May 2020 (12). Besides, a retrospective international cohort study which examined the differences in hospital emergency psychiatric presentations for NSSI during the COVID-19 pandemic, had observed an increase in the number of adolescents seeking emergency services owing to self-harm (13).

There are many causes of NSSI, and childhood trauma as a prominent traditional risk factor has been extensively studied (14, 15). Childhood trauma mainly refers to emotional and physical abuse or neglect from family or society, approximately one-third of children are reported to have experienced severe childhood trauma (16). Actual or potential physical harm to a child caused by a caregiver or other person using rude and inappropriate behavior toward the child, or chronic and inappropriate emotional reactions to the child, such as malicious rejection, intimidation, or use of sarcastic, insulting, and discriminatory language toward the child. There was also the possibility of being molested as a child, or even being coerced into doing something sexual. Numerous studies have shown that childhood trauma can lead to extensive adverse and lasting effects, such as chronic fatigue syndrome (17), poor academic achievements (18), adulthood depression and anxiety symptoms (19), and early drug use (20). Childhood trauma undermines children's ability to develop positive adaptations, and in turn, the vulnerability of children's adaptive resources leads them to adopt flawed alternative regulatory and relational strategies such as NSSI (21). Trauma experts have noted that, while individuals report NSSI for a variety of reasons, the most salient is reacting to perceived uncomfortable and overwhelming emotions (21). NSSI was conceptualized as being associated not only with intrapersonal motivations (e.g., management of internal states), but also with interpersonal motivations (e.g., to evoke others' emotional responses, such as pity or anger) (22). Child maltreatment is an urgent public health problem that deserves more attention and scientific investment during health and socioeconomic crises like COVID-19 (23). The recession, especially parental job loss, exacerbated the risk of child abuse (24).

In recent years, the mechanisms between childhood trauma exposure and NSSI behaviors have aroused great interest among psychological workers. For screening and preventing NSSI among adolescents, there is an urgent need for an efficient and operable tool. Previous research ideas have focused on a specific point, with the main relevant concepts being borderline personality, alexithymia, and resilience (25–27). However, it is not realistic to screen for everything in practice. In view of the above reasons, this study will focus on a comprehensive concept of psychological sub-health state (PSH). PSH is a psychological state between health and disease, which is characterized by emotional and behavioral problems and the decline of social adaptability, but it is not in line with clinical or subclinical disease diagnosis (28). PSH is generally regarded as a series of suboptimal health states, mainly manifested by unexplained mental fatigue, panic, anxiety, low self-esteem, nervousness, recklessness, and even suicidal thoughts (29). Adolescents in a PSH state may feel inexplicably restless and stressed, feeling that others are not being friendly to them, or having difficulty controlling their anger and impulsiveness. PSH usually does not seriously impact their study and life, but this kind of state can bring adverse psychological and behavioral consequences. This study would explore the pathway from previous adverse experience (childhood trauma), current psychological state (PSH state) to adverse outcomes (NSSI).

The primary objective of the current study was to investigate the prevalence of childhood trauma, PSH, and NSSI of Chinese adolescents during the COVID-19 pandemic. Additionally, we aimed to analyze the relationships between previous or undergoing childhood trauma, current detrimental PSH states and NSSI behaviors. More specifically, this study explored the risk factors for NSSI with the aim of reducing the incidence of NSSI in adolescents. Furthermore, we hypothesized that childhood trauma would be directly and indirectly related to NSSI via PSH. The structural equation model (SEM) would be applied to examine the mediating role of PSH in the relationship between childhood trauma and NSSI.

## Materials and Methods

### Participants

Participants were included in this survey if: (1) they were minors (<18 years old), and (2) they volunteered to participate in this survey. Adolescents with severe mental or physical illness, or with impaired audiovisual function would be excluded from the study. All participants were recruited from schools in Anhui Province, China, between October 2020 and April 2021. Firstly, under the guidance of their school teachers, we explained the purpose and process of the study to the participants and made sure that they fully understood the study. Written informed consent were then obtained from the participants and their guardians (parents, or other caregivers). This survey was approved by the Ethics Committee of Chaohu Hospital, Anhui Medical University (2019-kyxm-012). All research procedures were strictly in line with the principles of the Helsinki Declaration.

### Measures

A self-designed questionnaire was used to collect sociodemographic characteristics. All participants received a screening questionnaire for NSSI (30, 31), asking ‘Have you ever deliberately harmed yourself, but did not mean to kill yourself in the past year?’. Subsequently, a list of eight NSSI methods were presented as follows: (1) Have you ever hit yourself? (2) Have you ever pulled your hair yourself? (3) Have you ever banged your head or fisted against something? (4) Have you ever pinched yourself? (5) Have you ever scratched yourself? (6) Have you ever bitten yourself? (7) Have you ever exposed yourself to smoke, fire, flames, and overheated substances? and (8) Have you ever cut or pierced yourself? Participants who confirmed the reality that they had engaged in NSSI, the frequency of NSSI was investigated. The Cronbach's α coefficient for the NSSI was reported to be 0.776 (31).

The simplified version of the Childhood Trauma Questionnaire (CTQ-SF), a 28-item self-report questionnaire, was employed to assess a history of emotional and physical abuse and neglect that the participants had experienced or is experiencing. The original English version of the CTQ-SF was written by Professor Bernstein and his colleagues (32, 33) and was translated into Chinese by Zhao et al. (34). CTQ-SF was widely used in Chinese adolescents and proved to have good reliability and validity (35, 36). CTQ-SF consists of five subscales, including emotional abuse, emotional neglect, physical abuse, physical neglect, and sexual abuse.

The Multidimensional Sub-health Questionnaire of Adolescent (MSQA) was applied to assess participants' psychological health state. MSQA is a self-report symptom inventory developed for Chinese adolescents by Tao (37). MSQA consists of psychological health part and physical health part, and only the psychological health part was investigated in this study. psychological health part consists of 39 items, including 3 dimensions: emotional symptoms (17 items), behavioral symptoms (9 items) and social adaptation problems (13 items). Score points based on the duration of each symptom (1 point: none or last <1 week; 2 points: last ≥ 1 weeks; 3 points: last ≥ 2 weeks; 4 points: last ≥ 1 month; 5 points: last ≥ 2 months; 6 points: last ≥ 3 months). PSH state was determined if more than 7 items scored ≥4 points (38). MSQA has good reliability and validity (39), and Cronbach α coefficient is 0.96.

### Statistical Analysis

Data analyses were conducted with SPSS 21.0, and AMOS 21.0 software packages. Chi-square tests were performed on classified variables. The single-sample Kolmogorov-Smirnov test was used to verify the data distribution type. The independent sample *T-*test or Mann-Whitney U test were used to compare variables of NSSI group with the non-NSSI group. Spearman or Pearson correlation analyses were applied to describe the correlations among continuous variables. In addition, Binary Logistic regression was used to explore the risk factors of participants' NSSI.

We performed a structural equation model (SEM) to verify the hypothesis of the mediating effect of PSH in the relationship between childhood trauma and NSSI. Confirmatory factor analysis was conducted in AMOS 21.0, and generalized least squares method was employed for parameter estimation. PSH and NSSI were latent variables. PSH was constructed with emotional symptoms, behavioral symptoms, and social adaptation problems, and NSSI was constructed with NSSI (Yes or No) and NSSI frequency. The following goodness-of-fit measures were used to evaluate how well the hypothesized model fit the sample data: Chi-square degrees of freedom ratio (CMIN/DF), comparative fit index (CFI), goodness-of-fit index (GFI), adjusted goodness-of-fit statistic (AGFI), incremental fit index (IFI), normed fit index (NFI), relative fit index (RFI), Tacker-Lewis index (TLI), and root mean square error of approximation (RMSEA). Generally, a well-fitting model is indicated when CFI, GFI, AGFI, IFI, NFI, RFI, and TLI values are above 0.9, CMIN/DF is below 2, and the RMSEA value is below 0.08. All statistical significance was set at *p* < 0.05, and all reported *p*-values were bilateral.

## Results

### Social Demographic Characteristics of Participants

Based on the inclusion and exclusion criteria, we ultimately recruited 823 participants. After screening, 45 participants submitted incomplete questionnaires (33 for CTQ-SF questionnaires and 12 for MSQA questionnaires), thus, 778 questionnaires were eligible.

The demographic characteristics of NSSI group (*n* = 264, 33.9%) and non-NSSI group (*n* = 514, 66.1%) are shown in Table 1. And 72.3% participants of the NSSI group had frequently hurt themselves (≥5 times a year). Of the 778 participants, there were 391 (50.3%) male and 387 (49.7%) female aged 10 to 17 years (mean age: 12.7 ± 1.3 years). If sorted by grades, there are 352 (45.2%) primary school students (grades 5, 6), and 426 (54.8%) middle school students (grades 7, 8, 9, 10, 11). Generally, participants of younger or lower grades were more likely to develop NSSI behavior (*p* = 0.01). Among the 107 (13.8%) boarding students, 42.1% of them had NSSI, and the incidence of NSSI was not significantly higher than that of commuting students (*p* = 0.06). Additionally, nearly half of the participants (41.6%) had ever been or currently were left-behind children (i.e., children whose parents work in other cities and have been separated from their parents for more than half a year), and the incidence of NSSI was significantly higher than those who were accompanied by their parents (*p* = 0.02). Besides, most of the participants' parents were not well-educated (<9 years) and didn't have siblings. Additionally, 82.8% of the participants' parents are in married status, 15.7% of the participants' parents have divorced, and unfortunately 1.5% of the participants' parents were deceased. Over all, the presence of siblings, parents' marital state and education didn't differ significantly between the NSSI group and the non-NSSI group, while participants adolescents who were left-behind children or in primary schools were more likely to engage in NSSI.

**Table 1:** Social demographic characteristics of adolescents.

**Variables**	**Total participants**	**NSSI**		**Z/χ^2^**	***p***
	**(*n* = 778)**	**Yes (*n* = 264, 33.9%)**	**No (*n* = 514, 66.1%)**		
Age	12.68 (1.34)	12.53 (1.35)	12.75 (1.32)	−2.54	0.01
Gender				2.15	0.14
Male	391 (50.3%)	123 (46.6%)	268 (52.1%)		
Female	387 (49.7)	141 (53.4%)	246 (47.9%)		
Grade				6.34	0.01
primary school	352 (45.2%)	136 (51.5%)	216 (42.0%)		
middle school	426 (54.8%)	128 (48.5%)	298 (58.0%)		
Accommodation type				3.65	0.06
Boarding student	107 (13.8%)	45 (17.0%)	62 (12.1%)		
Commuting student	671 (86.2%)	219 (83.0%)	452 (87.9%)		
Father's educational level				0.83	0.36
<9 years	463 (59.5%)	163 (61.7%)	300 (58.4%)		
≥9 years	315 (40.5%)	101 (38.3%)	214 (41.6%)		
Mother's educational level				0.38	0.54
<9 years	486 (62.5%)	161 (61.0%)	325 (63.2%)		
≥9 years	292 (37.5%)	103 (39.0%)	189 (36.8%)		
Left behind status				5.75	0.02
Yes	366 (47.0%)	140 (53.0%)	226 (44.0%)		
No	412 (53.0%)	124 (47.0%)	288 (56.0%)		
Siblings				0.37	0.55
Yes	324 (41.6%)	106 (40.2%)	218 (42.4%)		
No	454 (58.4%)	158 (59.8%)	296 (57.6%)		
Parents' marital status				1.86	0.40
Married	644 (82.8%)	212 (80.3%)	432 (84.0%)		
Parental divorce	122 (15.7%)	48 (18.2%)	74 (14.4%)		
Death of a parent	12 (1.5%)	4 (1.5%)	8 (1.6%)		
NSSI frequency					
<5 times a year	N/A	73 (27.7%)	N/A	N/A	N/A
≥5 times a year	N/A	191(72.3%)	N/A		

*Values are presented as number (%) or mean (standard deviation)*.

Statistical description and comparison of childhood trauma and PSH among NSSI group and non-NSSI group.

The Mann–Whitney *U-*tests were used to compare childhood trauma and PSH between NSSI group and non-NSSI group (Table 2). More than half of the participants (56.2%) had moderate to severe childhood trauma and 43.7% of them had ever deliberately hurt themselves within the last year, which significantly exceeded that of participants without childhood trauma (21.4%). Furthermore, participants with NSSI behaviors scored much higher on each subscale of CTQ-SF than those without NSSI behaviors (*p* < 0.001). In addition, participants with PSH state account for only 26.1% and the incidence of NSSI was as high as 63.3%, while the incidence of NSSI among participants without PSH state is only 23.5%. The NSSI group scored significantly higher in emotional symptoms, behavioral symptoms, and social adaptation problems than the non-NSSI group (*p* < 0.001).

**Table 2:** The prevalence and intergroup comparison of childhood trauma and psychological sub-health state.

**Variable**	**Total sample**	**NSSI**		**Z/χ^2^**	***p***
	**(*n* = 778)**	**Yes (*n* = 264)**	**No (*n* = 514)**		
Childhood trauma	41.07 (11.98)	45.94 (12.81)	38.57 (10.71)	−8.59	<0.001
Yes	437 (56.2%)	191 (72.3%)	246 (47.9%)		
No	341 (43.8%)	73 (27.7%)	268 (52.1%)		
Emotional abuse	7.66 (3.49)	9.09 (4.00)	6.92 (2.94)	−9.18	<0.001
Physical abuse	6.40 (2.89)	7.13 (3.44)	6.03 (2.49)	−6.44	<0.001
Sexual abuse	5.80 (2.49)	6.24 (3.03)	5.58 (2.13)	−4.46	<0.001
Emotional neglect	11.72 (4.75)	13.07 (5.12)	11.03 (4.35)	−5.15	<0.001
Physical neglect	9.49 (3.25)	10.42 (3.40)	9.01 (3.06)	−5.47	<0.001
PSH state				107.44	<0.001
Yes	203 (26.1%)	129 (48.9%)	74 (14.4%)		
No	575 (73.9%)	135 (51.1%)	440 (85.6%)		
Emotional symptoms	33.01 (17.20)	43.87 (19.79)	27.43 (12.46)	−12.64	<0.001
Behavioral symptoms	17.81 (10.23)	23.88 (11.78)	12.7 (7.66)	−12.08	<0.001
Social adaptation problems	25.08 (12.36)	31.51 (14.07)	21.77 (9.88)	−10.56	<0.001

*Values are presented as number (%) or mean (standard deviation)*.

### Associations Between Childhood Trauma and Current PSH State

Table 3 presented the results of Spearman correlation analysis between the scores of CTQ-SF and MSQA. The total score of CTQ-SF was positively correlated with emotional symptoms (*r* = 0.42, *p* < 0.01), behavioral symptoms (*r* = 0.42, *p* < 0.01) and social adaptation problems (*r* = 0.41, *p* < 0.01). Also, high correlations were found between the PSH subscales (all *r* > 0.40, all *p* < 0.01).

**Table 3:** Associations between childhood trauma and psychological sub-health state (*n* = 778).

**Variable**	**1**	**2**	**3**	**4**	**5**	**6**	**7**	**8**	**9**
Childhood trauma	1.00								
Emotional abuse	0.68^**^	1.00							
Physical abuse	0.56^**^	0.48^**^	1.00						
Sexual abuse	0.42^**^	0.31^**^	0.40^**^	1.00					
Emotional neglect	0.81^**^	0.41^**^	0.31^**^	0.19^**^	1.00				
Physical neglect	0.73^**^	0.32^**^	0.23^**^	0.23^**^	0.47^**^	1.00			
PSH state									
Emotional symptoms	0.42^**^	0.48^**^	0.25^**^	0.17^**^	0.26^**^	0.30^**^	1.00		
Behavioral symptoms	0.42^**^	0.49^**^	0.26^**^	0.20^**^	0.24^**^	0.31^**^	0.82^**^	1.00	
Social adaptation problems	0.41^**^	0.44^**^	0.27^**^	0.20^**^	0.25^**^	0.31^**^	0.80^**^	0.77^**^	1.00

*Spearman correlation was used for statistical analysis*;

** *p < 0.01*.

### Risk Factors for NSSI

Binary Logistic regressions with the “Enter” method were applied to explore the risk factors of NSSI. As shown in Table 4, childhood trauma (OR = 2.20, CI = 1.56–3.10), PSH (OR = 5.15, CI = 3.58–7.40) and grade (OR = 1.90, CI = 1.35–2.68) were risk factors for NSSI (R^2^ = 0.23). According to the analysis results, the risk of NSSI was 1.9 times higher for primary school students than for middle school students, 2.2 times higher for participants with childhood trauma than for those without childhood trauma, and 5.15 times higher for those in PSH state than for those in full mental health.

**Table 4:** Risk factors for NSSI (*n* = 778).

	**B**	**SE**	**Wals**	***P***	**OR**	**95% CI**	
						**Lower**	**Upper**
Left behind	0.19	0.18	1.18	0.28	1.21	0.86	1.71
Grade	0.64	0.18	13.43	<0.01	1.90	1.35	2.68
PSH state	1.64	0.19	78.00	<0.01	5.15	3.58	7.40
Childhood trauma	0.79	0.18	20.11	<0.01	2.20	1.56	3.10
Constant	−2.02	0.20	103.80	<0.01	0.13		

*The statistical analysis used binary logistic regression with the “Enter” method; CI, confidence interval; OR, odds ratio; SE, standard error; R^2^ = 0.23; Grade, 1: primary school, 0: middle school; PSH, psychological sub-health*.

### Analysis of the Structural Equation Model

**Figure 1:**
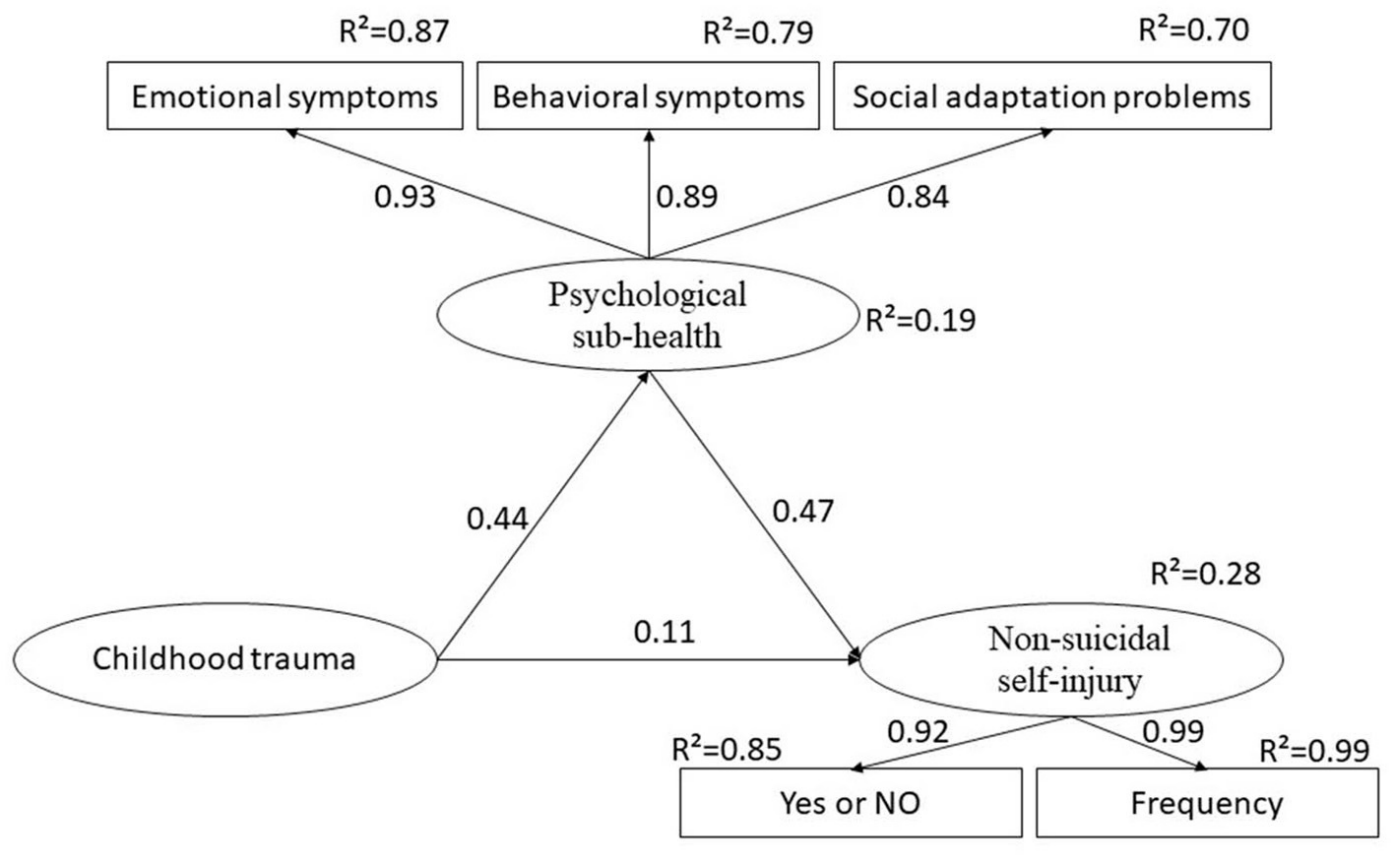
Structural equation model for all the participants. The numbers beside the arrows indicate standardized path coefficients. *R*^2^ represents squared multiple correlations. Probability level = 0.512; goodness-of-fit indices: CMIN/DF = 0.892; GFI = 0.997; AGFI = 0.992; NFI = 0.991; RFI = 0.980; IFI = 1.00; TLI = 1.00; CFI = 1.00; RMSEA < 0.001.

The SEM was successfully built (*p* = 0.512). Figure 1 presented the final results. Meanwhile, goodness-of-fit indices indicated satisfactory fit of the default model (CMIN/DF = 0.892; GFI = 0.997; AGFI = 0.992; NFI = 0.991; RFI = 0.980; IFI = 1.00; TLI = 1.00; CFI = 1.00; RMSEA < 0.001). Table 5 showed the direct, indirect, and total effects of the final SEM, and all values were standardized. There were direct effects of childhood trauma (β = 0.11, *p* < 0.001) and PSH (β= 0.47, *p* < 0.001) on NSSI. The indirect effects of childhood trauma on NSSI were 0.204 (*p* < 0.001), and the total effects were 0.317 (*p* < 0.001). In general, it was estimated that the predictors of NSSI (childhood trauma and PSH) could explain 28 percent of its variance (*R*^2^ = 0.28).

**Table 5:** Direct, indirect, and total effects of the final structural model.

**Endogenous variable**	**Exogenous variable**	**Direct effect**	**Indirect effect**	**Total effect**	**SMC**
PSH state	Childhood trauma	0.44^***^		0.44^***^	0.19
NSSI	Childhood trauma	0.11^***^	0.20^***^	0.32^***^	0.28
	PSH state	0.47^***^		0.47^***^	

*Standardized coefficient estimates are presented; SMC, Squared multiple correlations*;

*** *p < 0.001*.

## Discussion

PSH has been confirmed to have partial mediating effects between childhood trauma and NSSI. To the best of our knowledge, this is the first study to investigate the effect of childhood trauma exposure on the occurrence of NSSI behaviors from the perspective of current PSH state.

Our survey results showed that 33.9% participants had engaged in NSSI in the past year. 56.2% of the participants had moderate to severe childhood trauma, and 26.1% of the participants had PSH. Additionally, adolescents who are left-behind children and in lower grades were more likely to engage in NSSI. Most importantly, the current findings highlighted that childhood trauma exposure and current PSH state significantly increase the risk of developing NSSI behaviors. Specifically, childhood trauma and the current adverse PSH in adolescents can increase the NSSI risks by 2 times and 5 times, respectively. Meanwhile, the SEM successfully verified our initial hypothesis that childhood trauma positively predicted NSSI both directly and indirectly through PSH.

This paper explored whether the prevalence of NSSI among Chinese adolescents increased during the COVID-19 pandemic. As we predicted, the prevalence of NSSI had increased sharply. The prevalence rate of NSSI in this study is consistent with that in the COVID-19 epidemic period in the domestic and overseas, and higher than that before the COVID-19 epidemic. Consistent with our findings, an online survey shows that the proportion of Canadian adolescents who intentionally hurt themselves seems to be higher than before the COVID-19 pandemic (40). However, a recent meta-analysis of the prevalence of self-harm (including NSSI and suicide attempts) in infectious disease epidemics, mainly including epidemics of Spanish Flu, severe acute respiratory syndrome, Ebola virus disease and COVID-19, hadn't found strong evidence on the association of infectious epidemics with self-harm (41). However, the authors above also noted that the prevalence of self-harm may be underestimated due to lockdown, under-reporting, and poor-quality studies. Therefore, the impact of epidemic on NSSI needs to be confirmed by further accumulation of more evidence.

Our study found that prevalence of NSSI was higher in primary school students than in middle school students. A recent meta-analysis showed that the prevalence of self-injurious behavior peaked at age 15 and then decreased in females, but there is a paucity of data from studies of males (42). Studies have shown that the onset of engaging self-harm (regardless of suicidal intent) behavior typically between the ages of 12 and 15 years, and cease within 5 years of the initial onset (43, 44). The fact that we investigated the incidence of NSSI in the past year rather than the overall incidence may explain the apparently high rate of self-injury in lower grade participants. The neurodevelopmental vulnerability of early adolescents predisposes them to the development of emotional disorders and increased risk-taking behaviors (44). Additionally, our study found that left-behind children are more likely to injure themselves than those accompanied by their parents. Left-behind children are defined as minors whose parents have migrated to other cities to work or one of them has gone out to work and the other one has no ability to supervise them. Previous studies have demonstrated that children separated from their parents due to parental work, incarceration or abandonment show an increased likelihood of self-injury (45, 46). Therefore, child welfare institutions should pay more attention to left-behind children.

We investigated the childhood trauma of the participants, and the results showed that most participants had at least one type of childhood trauma, and childhood trauma exposure significantly increased the risk of NSSI. The strong link between NSSI and childhood trauma, especially sexual abuse, has been repeatedly confirmed and replicated by researchers (47). Despite the debatable direct causality of childhood trauma over NSSI, numerous studies have identified potential mediators of the relation between childhood trauma and NSSI. Of the proposed explanations of NSSI, affect dysregulation, dissociation, alexithymia, borderline personality had garnered broad empirical support (47). In our study, PSH was introduced as a new concept to explore the relationship between childhood trauma and NSSI. A PSH state is a mixture of psychological imbalances (e.g., restlessness, fatigue), behavioral symptoms (e.g., tantrums), and social maladjustment (e.g., reluctance to attend school). PSH state in adolescents are often overlooked because they do not appear to be severe enough, yet they are on the verge of psychological breakdown. In this study, we had assessed the current PSH state of participants, the results showed that one in four participants was in PSH state, and the PSH state sharply increased the risks of NSSI. Moreover, the SEM verified that childhood trauma has a direct impact on NSSI and could also indirectly increase NSSI through PSH state.

Adolescents in a PSH state are prone to feeling unmotivated throughout the day and night, and sleepiness makes it difficult for them to concentrate on their studies, yet childhood trauma has been shown to increase the risk of sleep disorders (36). Also, some meta-analyses demonstrated that sleep problems such as short sleep duration, sleep disorders and poor sleep quality are associated with NSSI and suicidal thoughts (48, 49). Additionally, adolescents in PSH are susceptible to anger and even impulsive behavioral problems, and research suggests that traumatic childhood experiences can weaken impulse control and lead to impulsive behavior (50). Impulsivity is thought to be a contributing factor to NSSI and is associated with the severity of NSSI (51). Besides, adolescents who experience childhood trauma exposure are more sensitive to stress (52) and they show more avoidance, emotional suppression, and negative emotional expression in response to stressful events (53). Moreover, most adolescents in a psychological subhealth state are reluctant to ask for help when they encounter difficulties, and are even unwilling to go to public places or participate in group activities (54). Previous study had found a positive correlation between the severity of social anxiety symptoms and the amount of childhood trauma exposure (55). NSSI was significantly associated with shame and feelings of inferiority (low social rank), and difficulties with interpersonal functioning may be a potential pathway for increased suicidal attempt due to social anxiety (56, 57). In addition, NSSI, suicide attempts, and suicidal ideation have not only complex psychological mechanisms, but the underlying biological factors have also been continuously reported (58). Hypothalamic-pituitary-adrenal (HPA) was proved to be a key moderator of childhood trauma exposure and adolescent mental health, with abnormalities in the HPA axis linking childhood trauma to a range of adverse psychological outcomes (59, 60). Childhood trauma and the HPA axis have been suggested to play a major role in the etiology of NSSI, and adolescents engaging in NSSI, particularly in those with a history of childhood trauma, exhibited significantly higher cortisol awakening responses (61). Furthermore, childhood trauma appeared to be genetically associated with undesirable behaviors such as self-injury and addiction, and they may share common genetic etiology (62, 63).

Despite the advantage and implications of this study, the results were still limited by methodological factors. First, this study used a cross-sectional design, therefore, causal inference is not acceptable. To confirm causality, prospective studies with staged sequential assessments of PSH state and the onset of NSSI behaviors would be informative. Second, our study sample included only Chinese adolescents, thus, the findings may not generalize well to other countries or other cultural contexts.

Third, considering that all variables are subjectively evaluated, it is possible for participants to exaggerate or attenuate their impressions and evaluations of trauma exposure. Furthermore, the practical application of the current MSQA to predict NSSI is limited. Therefore, it is urgent to develop a more professional and operational evaluation tool.

## Conclusion

Childhood trauma and the current adverse PSH in adolescents can increase the risk of NSSI. PSH has been confirmed to have partial mediating effects between childhood trauma and NSSI. Additionally, left-behind adolescents and younger adolescents deserve more attention from clinicians and educators. The present study provided potent evidence from a large sample for the mediating role of current PSH state between childhood trauma and NSSI. Since childhood trauma is difficult to prevent and heal, from the perspective of psychopathological mechanism, PSH state can be applied as an intermediate pathway to block the progression of childhood trauma to NSSI; while from the perspective of practical application, PSH state can be used as an alternative option which can be expediently assessed and intervened. Timely reversal of PSH state to healthy state can effectively prevent the consequences of undesirable behaviors. Overall, our findings provide clear directions for further research and clinical work on the etiologies, risk assessment, and treatment of NSSI.

## Data Availability Statement

As this study is still ongoing, the raw datasets for the current study will not be available until the end of this research project. Please contact the first author (Cui Huang, huang_cui66@163.com) for raw data requests.

## Author Contributions

The manuscript was designed and written by CH and QY. Data was collected by CH, QY, MG, XS, MYa, SS, PC, MYe, RP, and RZ, analyzed by CH and KZ, and verified by XZ. All authors read and agreed to the final manuscript.

## Funding

This study was supported by the National Natural Science Foundation of China (81801341), China International Medical Exchange Foundation (Z-2018-35-2002), the Anhui Provincial Key R&D Programme (202004j07020030), and Interdisciplinary project of clinical and basic disciplines of Anhui Medical University (No. 2101025103).

## Conflict of Interest

The authors declare that the research was conducted in the absence of any commercial or financial relationships that could be construed as a potential conflict of interest.

## Publisher's Note

All claims expressed in this article are solely those of the authors and do not necessarily represent those of their affiliated organizations, or those of the publisher, the editors and the reviewers. Any product that may be evaluated in this article, or claim that may be made by its manufacturer, is not guaranteed or endorsed by the publisher.

## References

[1] Halicka J Kiejna A. Non-suicidal self-injury (NSSI) and suicidal: criteria differentiation. Adv Clin Exp Med. (2018) 27:257–61. 10.17219/acem/66353 29521070

[2] Swannell SV Martin GE Page A Hasking P St John NJ. Prevalence of nonsuicidal self-injury in nonclinical samples: systematic review, meta-analysis and meta-regression. Suicide Life-Threat Behav. (2014) 44:273–303. 10.1111/sltb.12070 24422986

[3] Lang J Yao Y. Prevalence of nonsuicidal self-injury in Chinese middle school and high school students: a meta-analysis. Medicine. (2018) 97:e12916. 10.1097/MD.0000000000012916 30335024 PMC6211880

[4] Ribeiro JD Franklin JC Fox KR Bentley KH Kleiman EM Chang BP. Self-injurious thoughts and behaviors as risk factors for future suicide ideation, attempts, and death: a meta-analysis of longitudinal studies. Psychol Med. (2016) 46:225–36. 10.1017/S0033291715001804 26370729 PMC4774896

[5] Hawton K Harriss L. Deliberate self-harm in young people: characteristics and subsequent mortality in a 20-year cohort of patients presenting to hospital. J Clin Psychiatry. (2007) 68:1574–83. 10.4088/JCP.v68n1017 17960975

[6] De Berardis D Fornaro M Valchera A Cavuto M Perna G Di Nicola M. Eradicating suicide at its roots: preclinical bases and clinical evidence of the efficacy of ketamine in the treatment of suicidal behaviors. Int J Mol Sci. (2018) 19:2888. 10.3390/ijms19102888 30249029 PMC6213585

[7] Bachmann S. Epidemiology of suicide and the psychiatric perspective. Int J Environ Res Public Health. (2018) 15:425. 10.3390/ijerph15071425 29986446 PMC6068947

[8] Klomek AB. Suicide prevention during the COVID-19 outbreak. Lancet Psychiatry. (2020) 7:390. 10.1016/S2215-0366(20)30142-5 32353271 PMC7185940

[9] Guan K Fox KR Prinstein MJ. Nonsuicidal self-injury as a time-invariant predictor of adolescent suicide ideation and attempts in a diverse community sample. J Consul Clin Psychol. (2012) 80:842–9. 10.1037/a0029429 22845782 PMC3458144

[10] Patton GC Coffey C Sawyer SM Viner RM Haller DM Bose K. Global patterns of mortality in young people: a systematic analysis of population health data. Lancet. (2009) 374:881–92. 10.1016/S0140-6736(09)60741-8 19748397

[11] Plener PL. COVID-19 and nonsuicidal self-injury: the pandemic's influence on an adolescent epidemic. Am J Public Health. (2021) 111:195–6. 10.2105/AJPH.2020.306037 33439716 PMC7811075

[12] McIntyre A Tong K McMahon E Doherty AM. COVID-19 and its effect on emergency presentations to a tertiary hospital with self-harm in Ireland. Irish J Psychol Med. (2021) 38:116–22. 10.1017/ipm.2020.116 32993833 PMC7711341

[13] Ougrin D Wong BH Vaezinejad M Plener PL Mehdi T Romaniuk L. Pandemic-related emergency psychiatric presentations for self-harm of children and adolescents in 10 countries (PREP-kids): a retrospective international cohort study. Eur Child Adolesc Psychiatry. (2021) 1–13. 10.1007/s00787-021-01741-6 33677628 PMC7937052

[14] Klonsky ED Moyer A. Childhood sexual abuse and non-suicidal self-injury: meta-analysis. Brit J Psychiatry. (2008) 192:166–70. 10.1192/bjp.bp.106.030650 18310572

[15] Brown RC Plener PL. Non-suicidal self-injury in adolescence. Curr Psychiatry Rep. (2017) 19:20. 10.1007/s11920-017-0767-9 28315191 PMC5357256

[16] Zhang S Lin X Yang T Zhang S Pan Y Lu J. Prevalence of childhood trauma among adults with affective disorder using the Childhood Trauma Questionnaire: a meta-analysis. J Affect Disord. (2020) 276:546–54. 10.1016/j.jad.2020.07.001 32871685

[17] Kempke S Luyten P Claes S Van Wambeke P Bekaert P Goossens L. The prevalence and impact of early childhood trauma in Chronic Fatigue Syndrome. J Psychiatr Res. (2013) 47:664–9. 10.1016/j.jpsychires.2013.01.021 23421962

[18] Larson S Chapman S Spetz J Brindis CD. Chronic childhood trauma, mental health, academic achievement, and school-based health center mental health services. J School Health. (2017) 87:675–86. 10.1111/josh.12541 28766317

[19] Huh HJ Kim KH Lee HK Chae JH. The relationship between childhood trauma and the severity of adulthood depression and anxiety symptoms in a clinical sample: the mediating role of cognitive emotion regulation strategies. J Affect Disord. (2017) 213:44–50. 10.1016/j.jad.2017.02.009 28189964

[20] Huang C Yuan Q Zhang L Wang L Cui S Zhang K. Associations between childhood trauma and the age of first-time drug use in methamphetamine-dependent patients. Front Psychiatry. (2021) 12:658205. 10.3389/fpsyt.2021.658205 33868060 PMC8044866

[21] Yates TM. The developmental psychopathology of self-injurious behavior: compensatory regulation in posttraumatic adaptation. Clin Psychol Rev. (2004) 24:35–74. 10.1016/j.cpr.2003.10.001 14992806

[22] Yates TM Carlson EA Egeland B. A prospective study of child maltreatment and self-injurious behavior in a community sample. Dev Psychopathol. (2008) 20:651–71. 10.1017/S0954579408000321 18423099

[23] Martinkevich P Larsen LL Græsholt-Knudsen T Hesthaven G Hellfritzsch MB Petersen KK. Physical child abuse demands increased awareness during health and socioeconomic crises like COVID-19. Acta Orthopaed. (2020) 91:527–33. 10.1080/17453674.2020.1782012 32573297 PMC8023935

[24] Lawson M Piel MH Simon M. Child Maltreatment during the COVID-19 pandemic: consequences of parental job loss on psychological and physical abuse towards children. Child Abuse Neglect. (2020) 110(Pt 2):104709. 10.1016/j.chiabu.2020.104709 32893003 PMC7472978

[25] McFetridge MA Milner R Gavin V Levita L. Borderline personality disorder: patterns of self-harm, reported childhood trauma and clinical outcome. BJPsych Open. (2015) 1:18–20. 10.1192/bjpo.bp.115.000117 27703718 PMC4995580

[26] Iskric A Ceniti AK Bergmans Y McInerney S Rizvi SJ. Alexithymia and self-harm: a review of nonsuicidal self-injury, suicidal ideation, and suicide attempts. Psychiatry Res. (2020) 288:112920. 10.1016/j.psychres.2020.112920 32279008

[27] Tian X Lu J Che Y Fang D Ran H He X. Childhood maltreatment and self-harm in Chinese adolescents: moderation and mediation via resilience. BMC Public Health. (2021) 21:1561. 10.1186/s12889-021-11605-y 34404376 PMC8371889

[28] Bi JL Chen J Sun XM Nie XL Liu YY Luo R. The development and evaluation of a sub-health self-rating scale for university students in China. BMC Public Health. (2019) 19:330. 10.1186/s12889-019-6650-3 30898160 PMC6429791

[29] Yang ZM Yang XB Huang L. [A literature review on the conceptual framework of sub-health]. Zhongguo Zhong xi yi jie he za zhi Zhongguo Zhongxiyi jiehe zazhi. (2010) 30:757–63. 20929139

[30] Wan YH Hu CL Hao JH Sun Y Tao FB. Deliberate self-harm behaviors in Chinese adolescents and young adults. Eur Child Adolesc Psychiatry. (2011) 20:517–25. 10.1007/s00787-011-0213-1 21866416

[31] Wan YH Xu SJ Chen J Hu CL Tao FB. Longitudinal effects of psychological symptoms on non-suicidal self-injury: a difference between adolescents and young adults in China. Soc Psychiatry Psychiatr Epidemiol. (2015) 50:237–47. 10.1007/s00127-014-0917-x 24974078

[32] Bernstein DP Ahluvalia T Pogge D Handelsman L. Validity of the childhood trauma questionnaire in an adolescent psychiatric population. J Ame Acad Child Adolesc Psychiatry. (1997) 36:340–8. 10.1097/00004583-199703000-00012 9055514

[33] Bernstein DP Stein JA Newcomb MD Walker E Pogge D Ahluvalia T. Development and validation of a brief screening version of the Childhood Trauma Questionnaire. Child Abuse Negl. (2003) 27:169–90. 10.1016/S0145-2134(02)00541-0 12615092

[34] Zhao XF Zhang YL Li LF Zhou YF Li HZ Yang SC. Reliability and validity of the Chinese version of childhood trauma questionnaire. Chin J Clin Rehabil. (2005) 9:105–7. 34929586

[35] He J Zhong X Gao Y Xiong G Yao S. Psychometric properties of the Chinese version of the Childhood Trauma Questionnaire-Short Form (CTQ-SF) among undergraduates and depressive patients. Child Abuse Negl. (2019) 91:102–8. 10.1016/j.chiabu.2019.03.009 30856597

[36] Xiao D Wang T Huang Y Wang W Zhao M Zhang WH. Gender differences in the associations between types of childhood maltreatment and sleep disturbance among Chinese adolescents. J Affect Disord. (2020) 265:595–602. 10.1016/j.jad.2019.11.099 32090782

[37] Tao FB Chuan-Lai HU Sun YH. The development and application of multidimensional sub-health questionnaire of adolescents (MSQA). Chin J Dis Control Prevent. (2008). 4:309–14.

[38] Xu H Wu X Wan Y Zhang S Yang R Wang W. Interaction effects of co-consumption of fast food and sugar-sweetened beverages on psychological symptoms: evidence from a nationwide survey among Chinese adolescents. J Affect Disord. (2020) 276:104–11. 10.1016/j.jad.2020.07.030 32697688

[39] Wang H Lei-Lei LI Hong XU. Evaluation of the multidimensional sub-health questionnaire of adolescents. Chin Gen Pract. (2011) 14:2933–6.

[40] Turner BJ Robillard CL Ames ME Craig SG. Prevalence and correlates of suicidal ideation and deliberate self-harm in Canadian adolescents during the coronavirus disease 2019 pandemic. Can J Psychiatry. (2021) 7:1–13. 10.1177/07067437211036612 34378420 PMC9065494

[41] Rogers JP Chesney E Oliver D Begum N Saini A Wang S. Suicide, self-harm and thoughts of suicide or self-harm in infectious disease epidemics: a systematic review and meta-analysis. Epidemiol Psychiatr Sci. (2021) 30:e32. 10.1017/S2045796021000354 33902775 PMC7610720

[42] Steinhoff A Ribeaud D Kupferschmid S Raible-Destan N Quednow BB Hepp U. Self-injury from early adolescence to early adulthood: age-related course, recurrence, and services use in males and females from the community. Eur Child Adolesc Psychiatry. (2021) 30:937–51. 10.1007/s00787-020-01573-w 32572615 PMC8140957

[43] Whitlock J Eckenrode J Silverman D. Self-injurious behaviors in a college population. Pediatrics. (2006) 117:1939–48. 10.1542/peds.2005-2543 16740834

[44] Hawton K Saunders KE O'Connor RC. Self-harm and suicide in adolescents. Lancet. (2012) 379:2373–82. 10.1016/S0140-6736(12)60322-5 22726518

[45] Wang Y Zhang M Chen H. Self-injury among left-behind adolescents in rural China: the role of parental migration and parent-child attachment. Front Psychol. (2018) 9:2672. 10.3389/fpsyg.2018.02672 30666226 PMC6330276

[46] Teicher MH. Childhood trauma and the enduring consequences of forcibly separating children from parents at the United States border. BMC Med. (2018) 16:146. 10.1186/s12916-018-1147-y 30131056 PMC6103973

[47] Lang CM Sharma-Patel K. The relation between childhood maltreatment and self-injury: a review of the literature on conceptualization and intervention. Trauma Violence Abuse. (2011) 12:23–37. 10.1177/1524838010386975 21288933

[48] Pigeon WR Pinquart M Conner K. Meta-analysis of sleep disturbance and suicidal thoughts and behaviors. J Clin Psychiatry. (2012) 73:e1160–7. 10.4088/JCP.11r07586 23059158

[49] Khazaie H Zakiei A McCall WV Noori K Rostampour M Sadeghi Bahmani D. Relationship between sleep problems and self-injury: a systematic review. Behav Sleep Med. (2021) 19:689–704. 10.1080/15402002.2020.1822360 32991212

[50] Richard-Lepouriel H Kung AL Hasler R Bellivier F Prada P Gard S. Impulsivity and its association with childhood trauma experiences across bipolar disorder, attention deficit hyperactivity disorder and borderline personality disorder. J Affect Disord. (2019) 244:33–41. 10.1016/j.jad.2018.07.060 30336349

[51] Maxfield BL Pepper CM. Impulsivity and response latency in non-suicidal self-injury: the role of negative urgency in emotion regulation. Psychiatr Q. (2018) 89:417–26. 10.1007/s11126-017-9544-5 29018995

[52] Rauschenberg C van Os J Cremers D Goedhart M Schieveld JNM Reininghaus U. Stress sensitivity as a putative mechanism linking childhood trauma and psychopathology in youth's daily life. Acta Psychiatr Scand. (2017) 136:373–88. 10.1111/acps.12775 28758672

[53] Gruhn MA Compas BE. Effects of maltreatment on coping and emotion regulation in childhood and adolescence: a meta-analytic review. Child Abuse Negl. (2020) 103:104446. 10.1016/j.chiabu.2020.104446 32200195 PMC12352122

[54] Zhuang Y Tao F Yao R Zhang Q Fu L Han H. [Study of influential factors about trait coping style and the correlation between it and anxiety and depression in different student groups in Bengbu district]. Wei sheng yan jiu. (2011) 40:489–91. 21861356

[55] Bruijnen C Young SY Marx M Seedat S. Social anxiety disorder and childhood trauma in the context of anxiety (behavioural inhibition), impulsivity (behavioural activation) and quality of life. South Afr J Psychiatry. (2019) 25:1189. 10.4102/sajpsychiatry.v25i0.1189 30899577 PMC6424538

[56] Gilbert P McEwan K Irons C Bhundia R Christie R Broomhead C. Self-harm in a mixed clinical population: the roles of self-criticism, shame, and social rank. Brit J Clin Psychol. (2010) 49(Pt 4):563–76. 10.1348/014466509X479771 20109278

[57] Buckner JD Lemke AW Jeffries ER Shah SM. Social anxiety and suicidal ideation: test of the utility of the interpersonal-psychological theory of suicide. J Anxiety Disord. (2017) 45:60–3. 10.1016/j.janxdis.2016.11.010 27940416 PMC5234685

[58] Orsolini L Latini R Pompili M Serafini G Volpe U Vellante F. Understanding the Complex of suicide in depression: from research to clinics. Psychiatry Investig. (2020) 17:207–21. 10.30773/pi.2019.0171 32209966 PMC7113180

[59] Nikkheslat N McLaughlin AP Hastings C Zajkowska Z Nettis MA Mariani N. Childhood trauma, HPA axis activity and antidepressant response in patients with depression. Brain Behav Immun. (2020) 87:229–37. 10.1016/j.bbi.2019.11.024 31794798 PMC7327513

[60] Kuhlman KR Geiss EG Vargas I Lopez-Duran N. HPA-axis activation as a key moderator of childhood trauma exposure and adolescent mental health. J Abnorm Child Psychol. (2018) 46:149–57. 10.1007/s10802-017-0282-9 28215023 PMC10588887

[61] Reichl C Heyer A Brunner R Parzer P Völker JM Resch F. Hypothalamic-pituitary-adrenal axis, childhood adversity and adolescent nonsuicidal self-injury. Psychoneuroendocrinology. (2016) 74:203–11. 10.1016/j.psyneuen.2016.09.011 27665080

[62] Hodgson K Coleman JRI Hagenaars SP Purves KL Glanville K Choi SW. Cannabis use, depression and self-harm: phenotypic and genetic relationships. Addiction. (2020) 115:482–92. 10.1111/add.14845 31833150

[63] Sanabrais-Jiménez MA Sotelo-Ramirez CE Ordoñez-Martinez B Jiménez-Pavón J Ahumada-Curiel G Piana-Diaz S. Effect of CRHR1 and CRHR2 gene polymorphisms and childhood trauma in suicide attempt. J Neural Transmission. (2019) 126:637–44. 10.1007/s00702-019-01991-4 30874897

